# Supplementary material for: Are carbon emissions trading and green financial instruments synergistic? -Comprehensive quantitative research based on content analysis
Source: PLoS One. 2024 Mar 7;19(3):e0298601. doi: 10.1371/journal.pone.0298601 (PMC10919601; doi:10.1371/journal.pone.0298601)
Supplement: S1 Data — (ZIP) [file pone.0298601.s001.zip › Data/地级市Z_F工作报告.zip!/║■▒▒/╩«╤▀╩╨/└┤╫╘╣1⁄2╓┌║┼í╛┬φ┐╦╩2╛▌═°í┐_2006.docx]

各位代表：

　　现在，我代表市人民政府向大会作报告，请各位代表连同《十堰市国民经济和社会发展第十一个五年规划纲要（草案）》一并审议，并请各位政协委员和其他列席会议的同志提出意见。

　　一、"十五"时期经济社会发展回顾

　　"十五"时期是不平凡的五年。在市委的正确领导下，全市上下以邓小平理论和"三个代表"重要思想为指导，坚持以科学发展观统领经济社会发展全局，抢抓机遇，扎实工作，奋力抵御各种自然灾害，有效抑制经济运行和社会发展中的各种不稳定因素，大力实施工业兴市、生态立市和人才强市战略，国民经济持续快速发展，社会事业全面进步，生态环境明显改善，人民生活水平不断提高，社会主义民主政治和精神文明建设取得新的成效。

　　（一）国民经济快速增长。

　　五年来，全市经济实力不断增强，"十五"计划的主要目标提前一年实现。初步核算，2005年全市生产总值303亿元，五年年均增长10.4%；财政收入31.3亿元，年均增长10.9%；全社会固定资产投资累计完成337亿元，年均增长22.8%。2002至2004年我市连续三年跻身"全国综合实力百强城市"。

　　农村经济全面发展。2005年六县（市）实现生产总值121亿元，年均增长8%；全市第一产业增加值36亿元，年均增长4.8%。八大特色产业基地发展到330万亩，其中连片基地达100多万亩，农业产业化经营企业达204家。开展农民技能培训5.7万人次，有50多万农村劳力外出务工。五年累计投入各类扶贫资金16.1亿元，基本完成首批283个重点贫困村建设任务，组织实施搬迁扶贫6万多人，减少贫困人口和低收入人口38.9万人。解决了42.4万人饮水困难，我市被国家水利部授予"全国农村饮水解困先进集体"称号。

　　工业经济实力进一步增强。2005年规模以上工业实现增加值122亿元，年均增长16%。汽车、医药、电力、冶金工业增加值年均分别增长15%、26%、13.5%、88%。累计完成技术改造投资91亿元，比"九五"期间增长142%。丹江电解铝、农夫山泉、清大药业、渝安微型车等重大项目建成投产。十堰高新区实现工业增加值16亿元，年均增长23.4%。

　　第三产业发展水平明显提升。2005年第三产业实现增加值116亿元，年均增长11.2%。旅游产业长足发展，五年来，累计接待国内外游客2250万人次，实现旅游总收入91.7亿元，创汇2176万美元，均比"九五"期间增长2倍以上，我市荣获中国优秀旅游城市称号，武当山被评为全国风景名胜区示范点和首批4A级旅游区。商贸流通日趋活跃，2005年实现社会消费品零售总额125亿元，年均增长12.2%；大型连锁超市发展到53家，年营业额超过30亿元；中国（十堰）汽配城年销售额超过40亿元，汽配广场物流中心建成运营。信息服务业迅猛发展，建成了光纤骨干传输网和宽带城域网，固定电话用户达到55万户，移动电话用户突破70万户。房地产业发展迅速，累计完成房地产开发投资31亿元，开发各类商品房320万平方米，年均分别增长30%和20%。

　　财政金融运行平稳。2005年，全市财政收入突破30亿元大关，其中地方一般预算收入11.4亿元，年均可比增长13.7%，实现了财政收支的分年分级平衡。2005年末，全市金融机构各项存款余额406亿元，年均增长17%；各项贷款余额270.5亿元，年均可比增长12.5%。

　　（二）改革开放取得新突破。

　　各项改革深入推进。第二轮1023家国有、集体所有制企业改制任务基本完成，我市率先在全国实现了城市公交整体民营化；全力支持中央、省驻市企业分离办社会职能。财政管理体制改革稳步推进。进一步深化农村税费改革，免征农业税、特产税，取消不合理收费项目，依法完善土地二轮延包，乡镇综合配套改革取得阶段性成果。鼓励、支持和引导非公有制经济发展，全市个体私营企业发展到7.2万户，从业人员21.7万人，注册资金71.1亿元，比2000年分别增长18.4%、63.1%和430.5%。

　　对外开放水平不断提高。五年来，实际利用外资1.2亿美元，比"九五"期间增长1.6倍；利用市外境内资金80.1亿元，增长7倍。累计实现外贸出口2.5亿美元，比"九五"期间增长2倍。十堰海关正式开关。

　　（三）基础设施不断完善。

　　交通建设突飞猛进。"十五"期间全市公路建设养护总投资67.4亿元，比"九五"期间增长6倍；公路通车总里程达到8800多公里，比"九五"末增长15.8%，其中二级公路1467公里，增长2.6倍。汉十高速公路全线开通，十漫高速公路顺利推进。郧庙路、十巫路、316国道十白段和鲍竹路改造顺利完成，丹江口二桥建成通车。全市100%的乡镇、98%的行政村基本通公路。

　　水电建设取得历史性突破。黄龙滩扩机、陡岭子、孙家滩等22处水电工程建成投产，新增水电装机48万千瓦；丹江口、柏林220千伏输变电工程建成并投入运行，城乡电网改造全面完成。

　　城镇化步伐明显加快。2005年全市城镇化水平达到34.5%，比2000年提高6.7个百分点。建成了北京路、东风大道，综合改造了十堰、武当山火车站和人民路、公园路等23条道路，完成了市热电厂"四炉两机"工程，建成了神定河污水处理厂、刘家沟垃圾处理场。我市顺利通过国家园林城市和省级卫生城市复查，并连续三届获得省"楚天杯"奖。

　　（四）社会事业全面进步。

　　科技、教育事业快速发展。"十五"期间，全市科技总投入12.3亿元，组织实施各类科技项目1600多项，其中国家"863"计划和火炬计划项目6个，地膜水稻、常差速常互锁传动装置技术等一批科技成果达到国内领先水平，申报专利1550件。我市被科技部批准为全国农村信息化科技试点市，十堰高新区被批准为国家火炬计划汽车关键零部件产业基地。全市小学和初中适龄儿童入学率分别达到100%和96%，高考上省线人数逐年增加，中职教育资源整合初见成效，高等教育办学水平明显提高。积极培养、引进人才，广泛开展了人才共建工作。

　　文化体育、医疗卫生、广播电视事业蓬勃发展。成功举办了武当山、南水北调特种邮票首发式和武当国际旅游节，启动了武当山世界文化遗产保护日活动，成功承办省第十一届运动会。茅箭区五堰社区获得"全国体育先进社区"称号，郧县被命名为"全国文物工作先进县"。社区卫生服务业快速发展，农村合作医疗制度改革试点顺利推进，张湾、茅箭被评为全国社区卫生服务示范区。非典和禽流感防治取得胜利。广播、电视人口覆盖率分别达到90%和88%，基本实现了村村通广播电视。加大社会事业基础设施投入力度，建成了市体育中心、疾病预防控制中心和演艺大厅，改造了市图书馆、人民广场和传染病医院。

　　就业和社会保障成效显著。"十五"期间，全市新增就业15万人，安置下岗失业人员8万人，城镇登记失业率控制在4.3%以内。建立和完善了城市居民最低生活保障制度，7.8万城镇贫困居民纳入低保范围，基本实现应保尽保；实行农村特困户定期定量救助制度，6.7万人纳入农村贫困救助范围；稳步推进"福星工程"建设，五保对象基本实现了集中供养；战胜了特大洪灾等多种自然灾害，累计下拨救灾专项资金1.9亿元，妥善安置了灾区群众的生产生活。

　　社会保持稳定。加大社会治安综合治理力度，依法打击各类违法犯罪活动；积极排查和化解矛盾，集中处置群体性事件1500多件。我市被中央综治委授予"2001－2004年度全国社会治安综合治理优秀地市"，市公安局被评为"全国严打整治斗争先进集体"。建立安全生产责任制体系和应急反应机制，坚持开展安全生产专项整治活动，有效遏制了重特大事故的发生。强化食品、药品质量监管，保证了人民群众饮食和用药安全。

[FS:PAGE]

　　（五）人口资源环境协调发展。

　　加强人口和计划生育工作，人口自然增长率控制在6‰以内，符合政策生育率保持在95%以上，郧西县被授予全国"计生优质服务先进县"。大力节约资源，万元生产总值综合能耗比"九五"末下降22%；加强耕地保护，连续五年实现全市耕地总量动态平衡。加强南水北调中线工程核心水源区生态环境保护与建设，退耕还林195万亩，治理水土流失面积984平方公里，全市森林覆盖率51.6%；狠抓污染防治，关停污染企业96家，汉江干流十堰段、丹江口水库及堵河水质主要指标达到国家二类标准，在全市范围内建成了国家级生态示范区。

　　（六）人民生活水平稳步提高。

　　城乡居民收入稳定增长，2005年城镇居民人均可支配收入7200元，年均增长9.1%；农民人均纯收入1990元，年均增长6%。居民购买力显著增强，2005年居民人均储蓄存款6800元，比2000年增加3600元；居民人均消费水平为3800元，增加1300元。居民住房条件明显改善，城乡居民人均居住面积分别由2000年的14.7、26.6平方米提高到2005年的18.6、28.1平方米。

　　（七）精神文明和民主法制建设进一步加强。

　　民主建设继续推进。各级政府坚持定期向人大报告工作，向政协通报情况，重大决策征求人大、政协、民主党派、党外人士和人民团体意见，自觉接受法律监督、民主监督。五年来，市政府累计办理人大代表议案、建议937件，办理政协委员提案1555件，办复率100%。全市共受理群众信访2.3万件。高度重视基层民主建设，顺利完成了县市区换届选举和第五、第六届村（居）委会直选工作。

　　依法行政水平不断提高。深化行政审批制度改革，先后取消、调整行政许可和审批事项627项，市级许可和审批事项减少47.7%。深化执法体制改革，推行执法责任制、执法违法和错案追究制，强化执法监督。实施"四五"普法工作，成立行政服务中心，加强"市长公开电话"工作，推行政务公开，坚持开展民主评议政风行风、企业评议政府部门、社会公众评议政府机关等活动。加大审计监督力度，我市被授予全国"经济责任审计工作先进地区"称号。

　　精神文明建设向纵深推进。以创建国家级文明城市为动力，继续总结推广竹山"十星级文明农户"创建经验，积极开展系列创建活动，我市蝉联省级文明城市"四连冠"，2005年被评为全国文明创建工作先进城市，太和医院被命名为全国文明单位，市交通局、市审计局、十堰供电公司被命名为全国精神文明建设工作先进单位。深化全民国防教育，深入开展"双拥"活动，全面落实拥军优抚安置政策，积极支持部队和国防建设，我市实现国家和省"双拥模范城市"四连冠，并获得全国"人民防空先进城市"称号。

　　在三个文明建设中，外事、侨务、统计、民族宗教、地震、气象、水文、对台、老龄、残联、档案、地方志等方面工作都取得了新的成绩。

　　特别是刚刚过去的2005年，全市人民团结拼搏，开拓进取，沉着应对商用车市场疲软、产销滑坡的严峻形势，齐心协力抵御特大洪灾，积极防范禽流感疫情，努力化解经济和社会生活中出现的各种矛盾和不稳定因素，国民经济和各项社会事业平稳健康发展。全市生产总值同比增长4.7%，财政一般预算收入可比增长20%，全社会固定资产投资增长33.7%，社会消费品零售总额增长15%，直接利用外资可比增长86%，外贸出口增长66.4%，城镇居民人均可支配收入增长7.8%，农民人均纯收入增长3.9%。

　　这里着重报告去年"十件实事"的进展情况。在市三届人大一次会议上，我代表市政府郑重承诺，为全市人民着力办好"十件实事"。一年来，市政府把全面落实"十件实事"作为工作的重中之重，精心组织，克服种种困难，确保了"十件实事"的顺利推进。计划当年完成的安置就业、搬迁扶贫、农村贫困学生免费发放教科书和减免杂费、"福星工程"、免征农业税、"消茅工程"、新建沼气池和饮水工程、乡村油路建设等八件实事全部完成或超额完成目标任务；跨年度的城区四条道路和博物馆、游泳馆工程按年度计划已推进到位，今年可望全部建成。

　　各位代表，上述成绩的取得，是在市委的正确领导下，全市人民团结奋斗、辛勤劳动的结果，是历届政府打下良好基础和市人大、政协监督支持的结果。在此，我代表市政府，向所有参与、支持、关心十堰发展的同志们、朋友们表示崇高的敬意和衷心的感谢!

　　在总结成绩的同时，我们也清醒地认识到，我市经济社会发展中还存在不少问题和困难：二元经济结构矛盾突出，城乡发展差距较大；农业基础脆弱，农业增效、农民增收的长效机制尚未形成；经济增长的内生动力不足，有竞争力的企业和产品太少；就业和社会保障压力较大，部分下岗职工、城镇低收入居民和贫困地区群众生活还比较困难；政府工作中还存在很多缺点和不足，尤其是少数公务人员依法行政观念淡薄，还存在官僚主义、形式主义和腐败行为。这些问题和困难，需要在今后工作中认真加以解决。

　　二、"十一五"时期的基本思路和主要任务

　　今后五年是我市全面建设小康社会、构建和谐十堰的关键时期。这一时期，我市发展面临难得的机遇和有利条件：国家实施中部崛起战略、推进新阶段扶贫开发，东风公司实施中长期事业计划，南水北调中线工程和银沪高速公路、襄渝铁路二线等一批国家重点工程全面实施，汉江--堵河流域水能资源加快开发，汽车城、武当山、水源区三大品牌进一步整合和利用，这些将为十堰加快发展提供强大动力。同时，东风公司总部外迁带来的负面影响将逐渐显现，南水北调水源区环境保护要求更高、移民安置任务非常艰巨，我市也面临着严峻挑战。我们要紧紧抓住机遇，积极应对挑战，努力促进经济社会健康发展。

　　"十一五"时期我市经济社会发展的指导思想是：以邓小平理论和"三个代表"重要思想为指导，以科学发展观统揽经济社会发展全局，以全面建设小康社会、推进和谐十堰建设为目标，以改革开放和科技创新为动力，统筹城乡发展，转变经济增长方式，推进工业兴市、生态立市和人才强市，努力把十堰建成现代化的汽车城、水电城、旅游城、生态城和区域性中心城市。

　　未来五年经济社会发展的主要目标是：全市生产总值年均增长10%，地方一般预算收入年均增长10%，全社会固定资产投资年均增长16%，社会消费品零售总额年均增长10%，出口总额年均增长15%，利用外资年均增长10%，城镇居民人均可支配收入年均增长7%，农民人平纯收入年均增长5%，人口自然增长率控制在6‰以内，城镇登记失业率控制在5%以内，全市森林覆盖率达到56%，万元生产总值综合能耗比"十五"期末降低20%。

　　经过五年的不懈努力，我市发展可望实现六大突破：生产总值突破500亿元，财政收入突破50亿元，累计固定资产投资总额突破800亿元，社会消费品零售总额突破200亿元，利用外资突破1亿美元，城镇居民人均可支配收入突破1万元，农民生活水平也有较大提高。实现了上述目标，我市经济实力将跃上新台阶，社会发展将实现历史性跨越，城乡面貌将显著改观，群众生活质量将大幅提升。

　　（一）建设社会主义新农村。

　　积极发展现代农业。严格保护耕地，保持耕地动态平衡，加强农业基础设施建设，加快农业科技进步，提高农业综合生产能力。充分发挥比较优势，加快发展柑桔、中药材、有机茶、魔芋、食用菌、畜牧、蔬菜、黄姜等八大特色高效生态种养业，大力发展水产养殖业和烟叶、木瓜等种植业，形成现代农业产业集群。用产业化方式经营农业，培育1-2家收入过5亿元的国家级龙头企业，5-10家收入过亿元的省级龙头企业，打造一批名特优农产品品牌。努力提高农业市场化水平，形成农产品市场网络，发展各种形式的中介服务组织，健全农业社会化服务体系。

[FS:PAGE]

　　千方百计增加农民收入。大力发展县域经济，拓宽农民增收渠道。积极发展劳务经济，重点做好职业技能培训、集中输出和维权服务三个方面的工作，组织引导农村富余劳力向非农产业转移。加大新阶段扶贫开发力度，继续抓好941个重点贫困村的整村推进，加快实施《城区农村扶贫规划》，力争五年基本解决现有34万绝对贫困人口温饱问题，帮助低收入人口进一步改善生产生活条件。

　　大力发展农村公共事业。增加财政支农支出和社会帮扶投入，支持农村教育、卫生、文化等公共事业建设。加快农村学校布局调整步伐，继续实施农村中小学危房改造工程，认真落实国家对农村贫困学生的"两免一补"政策。全面实施第二轮农村初保规划，稳步推进以大病统筹为主的新型农村合作医疗，到2010年参保率达到90%以上。改善农民生产生活条件，供水、供电、广播、电视基本入户，新建和改造乡村道路1万公里，大力推进"一建三改"。

　　（二）大力推进新型工业化。

　　做强做大汽车及零部件产业。坚定不移地做好政企共建工作，全力服务东风公司实施中长期事业计划，积极争取东风公司在十堰多上新项目。以重型商用车、客车、特种专用车、微型车等项目建设为重点，发展整车生产。依托东风商用车生产基地，大力发展关键总成和具有比较优势的零部件，着力开发科技含量高的产品。支持市域内企业与国内外优势企业合资重组，加快创新体系建设，鼓励企业全方位开拓市场，建成国际化的商用车及零部件生产和技术创新基地。2010年各类整车生产规模达到55万辆以上，零部件达到60万辆份以上。

　　加快发展特色产业。科学有序开发水电产业，抓好潘口、孤山、小旋、龙背湾等水电站建设，力争2010年全市水电总装机达到300万千瓦以上；加快建设十襄、潘十等输变电工程，建成稳定可靠的电力生产供应网络。高起点发展生物医药产业，建设现代中药针剂生产基地。大力发展绿色食品产业，重点开发农夫山泉系列饮料、郧西山珍野葡萄酒、郧县白羽乌鸡胶囊、竹溪双竹魔芋面、房县南瓜、竹山葛根等特色产品。培育壮大矿产产业，重点抓好钒冶炼系列开发、金银矿开采加工和大理石、绿松石精加工等项目建设。积极发展信息制造业，大力开发汽车电子及汽车系统控制软件。

　　加强工业园区建设。支持十堰高新区加快发展，强化汽车关键零部件产业基地、高新技术产业孵化中心功能，更好地发挥对外开放的窗口作用和区域经济的龙头作用。县市区都要着重抓好开发区和工业园区的规划建设，发展特色产业，提高产业集约化水平和规模集聚效益，培育新的经济增长点。

　　（三）全面提升第三产业。

　　突破性发展旅游业。以武当山为龙头，以城区为中心，突出山、水、车特色，着力培育旅游品牌。加快旅游基础设施建设，重点实施城区游客中心、武当山旅游服务中心、五龙景区综合开发、玉虚宫维修等旅游项目。加快特色旅游产品开发，加强区域旅游合作，打造精品旅游线路。2010年全市接待国内外游客人数突破1000万人次，实现旅游总收入50亿元以上。

　　积极发展商贸物流业。扩大中国（十堰）汽配城市场规模，形成全国性的汽配集散中心。建设武当国际汽车商贸城、鄂西北小商品市场和粮油、蔬菜、农产品交易市场，推进"万村千乡"试点工程，加快汽配、日用工业品、农产品等物流中心建设，把十堰建设成为区域性物流中心。

　　加快发展新兴服务业。积极推进电子商务、电子政务和企业信息化建设，加快发展金融保险业和社区服务业，大力促进法律、会计、审计、咨询、资产评估等中介服务业发展，引导房地产业健康发展。

　　（四）加强交通和城镇建设。

　　全力推进交通建设。重点服务襄渝铁路二线、十漫高速公路建设，大力争取襄天、十房高速公路和丹江与汉十高速公路连接线一级路建设；继续实施二级公路骨架路网工程，市县公路全部达到二级以上标准，乡镇全部通达等级油路，村村通公路，80%以上的行政村通油（水泥）路。

　　加快城镇建设步伐。进一步加强城市和城镇规划管理工作。以建设区域性中心城市为目标，注重特色，科学布局，重点提升中部组团，积极发展东、西部组团，规划北向延伸，形成合理功能分区。拓展城区发展空间，优化城市形态。继续改善城区道路体系，续建天津路、浙江路、甘肃路、重庆路，新建中岳路、大连路、上海路、风神大道。加快各县市城区建设步伐，重点建设一批边贸型、基地型、旅游型等各具特色的小城镇，完善城镇体系。

　　（五）建设资源节约型、环境友好型社会。

　　提高资源利用效率。珍惜和合理利用国土资源，抓好汽车、建材等行业及重点企业的节能、节水、节材工作，加快农业节水工程建设，发展节能节地型公共建筑和住宅，降低单位产品能源消耗。组织实施重点区域、重点行业、重点企业循环经济试点，优先支持重大资源循环利用项目。

　　扎实做好移民安置和水源区生态环境建设工作。全力服务南水北调工程建设，做好被淹没城镇、企业、专业项目等迁建重建工作，积极争取国家政策支持和受水区对口支援；落实补偿补助政策，认真做好移民安置工作。继续抓好退耕还林、生态公益林、天然林保护等重点工程建设，治理水土流失面积2500平方公里。加大污染防治力度，抓好城区和各县市污水处理厂建设。

　　（六）深入推进改革开放。

　　深化经济体制改革。引导改制企业加快管理、分配、用工等内部制度改革，建立现代企业制度；完善国有资产监管体系，促进国有资产保值增值。在稳定家庭联产承包责任制的基础上，依法流转土地、山场承包经营权；巩固农村税费改革成果，深化乡镇综合配套改革，确保农民负担不反弹；推进农村信用社改革，建立和完善农业保险体系。继续深化部门预算、国库集中收付、政府采购和收支两条线管理制度改革，完善公共财政体系。

　　加快发展非公有制经济。坚决落实和完善以放宽准入、公平待遇为重点的各项政策措施，加强信息服务、技术服务和人才培训服务，完善信用担保体系，引导非公有制企业健康发展。

　　全方位扩大对外开放。立足特色资源和比较优势，不断优化环境，创新招商引资方式，提高利用外资的规模和水平。利用各种协作区平台，广泛开展横向经济联合；面向南水北调中线工程沿线城市及受水区，寻求新的经济技术合作。培植一批竞争力强的出口企业，加快外贸出口示范区和出口基地建设，扩大特色产品出口。

　　（七）大力实施科教兴市和人才强市战略。

　　切实推进科技创新。加强重点领域和关键技术的科技攻关，重点实施汽车及关键零部件、环境保护与南水北调中线水源区可持续发展、绿色食品与生态农业、生物技术与新医药、信息化等科技攻关专项计划。加强产学研合作和技术研发中心建设，大力发展科技中介服务，推进科技成果向现实生产力转化。

　　优先发展教育事业。全面推进素质教育，巩固"两基"成果，基本普及高中阶段教育。加快郧阳师专和十堰职业技术学院扩建步伐。扩大职业教育规模，实施学历证书与职业资格证书的"双证沟通"制度。大力发展民办教育，实现办学体制多元化。

　　深入实施人才强市战略。加强行政管理人才、企业经营管理人才和专业技术人才队伍建设，抓紧培养专业化高技能人才和农村实用人才。改善人才引进、使用环境，加大人才政策落实力度，健全人才评价、选拔任用和激励保障机制。规范人才市场管理，优化配置人才资源。

　　（八）努力构建和谐十堰。

　　做好就业和社会保障工作。大力拓宽就业渠道，健全就业服务体系，完善对困难群众的就业援助制度，力争每年新增就业3万人以上。进一步扩大社会保险覆盖面，加大社会保险基金征缴力度，逐步实现市级统筹。切实解决失地农民和受灾群众的生产生活困难。以城市低保、农村特困户、五保户等特困群体为主要救助对象，积极开展社会救助活动。

[FS:PAGE]

　　推进各项社会事业发展。加快市博物馆、美术馆、健身中心等文体设施建设，广泛开展群众性文体活动，努力提高竞技体育水平；深化文化体制改革，培植文化产业龙头企业；加强文物管理，净化文化市场。推进广播电视村村通工程，广播、电视人口覆盖率均达到90%以上。完善公共卫生体系，健全社区医疗服务网络与功能，提高快速应急处理能力和重大传染性疾病防治能力；加强环境卫生建设，强化卫生监督，认真解决群众看病难、看病贵问题。以稳定低生育水平、提高出生人口素质、改善出生人口性别比为重点，加快建立人口计生工作新机制，加强计生服务站建设。支持残疾人事业发展，保护妇女儿童合法权益，高度重视人口老龄化问题。大力推进和谐社区建设，提高社区服务功能。

　　保障人民群众生命财产安全。积极推进"平安十堰"建设，加强社会治安综合治理，认真排查并着力解决影响社会安定的突出问题，严密防范和依法严厉打击各种犯罪活动。强化安全生产责任，减少和防止重特大事故发生。深入开展整顿和规范市场经济秩序工作，切实维护消费者合法权益。强化对食品、药品、餐饮卫生等的监管，保障人民群众健康安全。建立健全应急管理体系，提高应对突发公共事件的能力。健全信访工作机制，畅通民情民意沟通渠道，正确处理人民内部矛盾。

　　（九）加强政治文明和精神文明建设。

　　加强精神文明建设。努力提高全市人民的文明素质和整个城市的文明程度，争创国家级文明城市。以社区创建为重点，推进城市文明创建活动；深入开展"十星级文明农户"创建活动，推进农村精神文明建设。加强全民国防教育，巩固军政军民团结，加强人民防空和民兵预备役建设，积极争创全国"双拥模范城市"五连冠。

　　加强民主法制建设。发展民主政治，坚持依法治市。认真执行人大及其常委会的决定决议，自觉接受人大监督，接受政协民主监督，接受新闻舆论监督和人民群众监督，主动加强同民主党派、工商联和社会各界的联系，充分发挥工会、共青团、妇联等人民团体的桥梁纽带作用，建立公众参与、专家论证和政府决策相结合的决策机制，推进决策的科学化、民主化、规范化。推进政务公开，切实做好财政与编制政务公开试点工作。发展基层民主，保证人民群众依法行使权利。深入贯彻落实国务院《依法行政实施纲要》，努力建设法治政府。加快建立权责明确、行为规范、运转协调、公正透明、监督有效的行政执法体制，保证法律法规正确实施。认真开展"五五"普法宣传教育，不断增强全社会的法治意识和遵法守法的自觉性。

　　加强勤政廉政建设。进一步转变政府职能，坚持运用经济、法律手段调节经济运行，把更多的公共资源投向社会管理和公共服务，用更多的精力推进社会发展和解决民生问题。建立健全公务员问责制度和科学的政绩考评机制，完善目标管理和重大事项督办制度，进一步转变机关工作作风，努力提高行政效能。全面落实党风廉政建设责任制，加强源头治理腐败工作，加大大案要案查处力度，严肃查处损害群众利益的案件，不断推动廉政法制建设和反腐败斗争深入开展。

　　三、扎实做好"十一五"开局之年的工作

　　2006年是实施"十一五"规划的第一年。全面完成"十一五"规划，今年起好头、开好局至关重要。今年全市经济社会发展的主要预期目标是：生产总值增长10%左右，全社会固定资产投资增长18%，财政一般预算收入增长10%，社会消费品零售总额增长12%，利用外资增长10%，外贸出口总额6000万美元，城镇居民人均可支配收入增长7%，农民人均纯收入增长6%，居民消费价格涨幅低于4%，城镇登记失业率、人口自然增长率控制在省定目标以内，万元生产总值综合能耗降低4%。"十一五"时期我市经济社会发展任务和工作措施，也包括了今年政府的各项工作。我们要按照"十一五"规划的总体部署，切实抓好今年各项目标任务的落实，突出抓好以下事关全局的重要工作。

　　（一）注重新农村建设，促进城乡协调发展。坚持规划先行，抓好试点示范，有步骤、有计划、有重点地逐步推进新农村建设，力争今年有良好开局。稳定提高粮食综合生产能力，实现一年受灾一年恢复，力争粮食总产达到80万吨以上。大力发展特色产业，新建特色板块基地30万亩，培植一批龙头企业，争取新认证绿色有机食品品牌20个以上。加强农村富余劳动力培训转移工作，争取今年再转移6万人以上。加强农村基础设施建设，新建沼气池1.5万口、渠道120公里，完成人工造林40万亩，恢复水毁饮水解困工程3000多处，解决3.6万人饮水安全问题，鼓励和支持农民投工投劳建设直接受益的农田水利设施。切实抓好重点贫困村、重点老区乡镇和库区建设，基本解决郊区农村行路难、通讯难问题。着力推进乡镇机构、农村义务教育和县乡财政体制改革，认真落实取消农业税政策，切实防止农民负担反弹，高度重视农村债务化解工作。坚持"工业反哺农业、城市支持农村"，建立以工促农、以城带乡的长效机制。

　　（二）注重园区建设，加快工业发展。积极鼓励和引导企业向工业园区集聚，培育新的经济板块，力争使园区成为市域经济的重要增长极。加快万向通达工业园、普林工业园、温州工业园等园区建设，促进产业集群发展。积极支持东风公司和市属骨干企业产品结构升级，扩大东风商用车、渝安微型车产量产能，支持三环专汽、神鹰、神河专用车发展。集中力量做好重点企业的改制重组工作，鼓励地方企业与东风公司及国内外大企业的合资合作，抓紧推进东风轮胎厂的政策性破产工作和华阳集团改制后的发展生产工作，抓好车城商场、市特种铸铁厂、市金亨合金公司等企业的依法破产工作，全面完成第二轮企业改制任务。

　　（三）注重培育消费热点，促进第三产业优化升级。完成全市水体旅游规划编制工作，推进武当山游客中心、文化广场、南岩景区游客步行通道等旅游重点项目建设，进一步改善旅游环境，延伸旅游产业链条，力争全年接待国内外游客和实现旅游收入增长10%以上。加强市区物流园区和配送中心系统建设，加快汽配城物流中心及汽车大市场、粮食现代物流系统等商贸流通项目建设。着力培育卫生保健、物业管理、家政服务等就业容量大、带动力强、与人民生活密切相关的社区服务业。大力发展文化体育产业，办好市第三届全民健身运动会。

　　（四）注重招商引资，加强重点项目建设。对今年计划安排的40个重点项目，实行项目责任制和首长负责制，进行跟踪服务和目标考核，确保顺利推进。切实做好东风公司ZD30发动机、D310车身、Y2越野车、微轿和大马力发动机等重大项目的服务工作。加快普林汽车零部件、先锋模具、清大药业二期、松树岭和鄂坪电站等项目建设，力争年内建成投产。继续推进万向通达汽车零部件、东风小康微型汽车、三里坪电站、竹山至竹溪输变电工程等续建项目建设。启动并推进周家垸、白沙河电站和柏林输变电增容工程等新建项目，力争潘口、孤山等电站尽快动工兴建。加大招商引资力度，精心组织第七届武当国际旅游节和京津地区对口扶持合作项目推介会，继续采取灵活多样的招商方式，引导市外境外资金投向支柱产业和重点项目。

　　（五）注重基础设施建设，提升城市功能。推进城区出口路和高速公路连接线工程建设，确保天津路、甘肃路、重庆路、浙江路如期竣工；加快十漫高速公路建设，基本完成房县城关至温泉一级公路改造，力争柳树垭公路隧道开工建设，加强省际出口路、经济断头路建设，提高对内对外连接的通达度。确保市博物馆、游泳馆今年竣工开馆，动工兴建行政中心，加快公交设施、停车场、集贸市场和城区公厕建设。开展城市环境综合整治，继续加强城市亮化、美化、绿化工程建设。积极稳妥地推进市政公用事业改革，加快城市供水、供热、环卫保洁和市政公用设施管护的市场化进程。

[FS:PAGE]

　　（六）注重生态环境建设，确保水源区生态安全。认真做好库区移民安置的试点示范，积极争取被淹没集镇迁建、污水处理厂、垃圾处理厂、小流域综合治理等一批项目年内开工建设。切实做好水源区的污染防治工作，启动实施"水污染防治规划"，治理水土流失面积200平方公里。加强对排污企业的监督管理，督促企业依法实施清洁生产，抓好工业废水、生活污水防治。加大神定河污染治理力度，推进水价改革，建设配套管网，促使污水处理厂正常运行。抓好黄姜加工企业污染治理，推广黄姜清洁生产新工艺，确保2007年6月30日前全部达标排放，逾期不能达标排放的企业一律关停。坚决制止乱开乱采矿产资源，防止无序开发造成生态环境新的破坏。严格实行项目核准制和备案制，对建设项目依法实行环保一票否决。

　　（七）注重民生问题，推进和谐十堰建设。认真解决群众生产生活中的实际问题，突出抓好城乡群众最为关心的十件实事。（1）高度重视就业和社会保障工作。确保全年城镇新增就业3.5万人、扩面新增参保6万人次。（2）改善城市居民生活环境。新建公厕10个，新增停车泊位2000个，新建或改造大型集贸和小商品市场3个。（3）加强农村富余劳动力转移培训工作。抓好"阳光工程"、扶贫培训、就业培训和实用技术培训，完成各类培训50万人次。（4）推进新阶段扶贫开发。减少贫困人口4万人、低收入人口5万人，完成扶贫搬迁和地质灾害搬迁1万人的任务。（5）做好农村危房改造和孤儿救助工作。改造危房2000户，将农村分散供养的孤儿全部纳入救助范围。（6）落实贫困学生资助政策。将农村义务教育阶段"两免一补"对象扩大到10万名以上。（7）努力缓解农民看病难、看病贵问题。重点加强10个乡镇卫生院和50个村卫生室建设，认真抓好新型农村合作医疗试点推广普及工作。（8）加强农村市场体系建设。推进"万村千乡"试点工程，重点建设20个乡镇超市和100个村级小超市。（9）推进农村交通建设。新建通村油路（水泥路）800公里。（10）改善城区交通状况。改造十堰火车站至白浪道路10公里，建设白浪至十漫高速公路许家棚出口一级路10公里。

　　各位代表!"十一五"时期，是我市经济社会发展的关键时期，做好今后五年的工作，对于加快十堰发展、全面建设小康社会，具有极为重要的意义。让我们高举邓小平理论和"三个代表"重要思想伟大旗帜，全面贯彻落实党的十六届五中全会、省委八届九次全会和市委二届十次全会精神，在市委的正确领导下，在市人大、政协的监督支持下，振奋精神，团结拼搏，锐意进取，乘势而上，为全面开创十堰改革发展的新局面而努力奋斗！
